# Geostatistical analysis of Malawi’s changing malaria transmission from 2010 to 2017

**DOI:** 10.12688/wellcomeopenres.15193.2

**Published:** 2019-07-04

**Authors:** Michael Give Chipeta, Emanuele Giorgi, Donnie Mategula, Peter M. Macharia, Chimwemwe Ligomba, Alinane Munyenyembe, James Chirombo, Austin Gumbo, Dianne J. Terlouw, Robert W. Snow, Michael Kayange

**Affiliations:** 1 Malaria Epidemiology Group, Malawi-Liverpool Wellcome Trust Research Programme, Blantyre, Malawi; 2 Lancaster Medical School, Lancaster University, Lancaster, LA1 4YW, UK; 3 Population Health Unit, Kenya Medical Research Institute - Wellcome Trust Research Programme, Nairobi, Kenya; 4 National Malaria Control Programme, Malawi Ministry of Health, Lilongwe, Malawi; 5 Liverpool School of Tropical Medicine, Liverpool, L3 5QA, UK; 6 Centre for Tropical Medicine and Global Health, Nuffield Department of Clinical Medicine, University of Oxford, Oxford, OX1 2JD, UK

**Keywords:** Model-based geostatistics, malaria, Malawi, Plasmodium falciparum

## Abstract

**Background: **The prevalence of malaria infection in time and space provides important information on the likely sub-national epidemiology of malaria burdens and how this has changed following intervention. Model-based geostatitics (MBG) allow national malaria control programmes to leverage multiple data sources to provide predictions of malaria prevalance by district over time. These methods are used to explore the possible changes in malaria prevalance in Malawi from 2010 to 2017.

**Methods:** *Plasmodium falciparum* parasite prevalence ( *Pf*PR) surveys undertaken in Malawi between 2000 and 2017 were assembled. A spatio-temporal geostatistical model was fitted to predict annual malaria risk for children aged 2–10 years ( *Pf*PR _2–10_) at 1×1 km spatial resolutions. Parameter estimation was carried out using the Monte Carlo maximum likelihood methods. Population-adjusted prevalence and populations at risk by district were calculated for 2010 and 2017 to inform malaria control program priority setting.

**Results: **2,237 surveys at 1,834 communities undertaken between 2000 and 2017 were identified, geo-coded and used within the MBG framework to predict district malaria prevalence properties for 2010 and 2017. Nationally, there was a 47.2% reduction in the mean modelled *Pf*PR _2-10 _from 29.4% (95% confidence interval (CI) 26.6 to 32.3%) in 2010 to 15.2% (95% CI 13.3 to 18.0%) in 2017. Declining prevalence was not equal across the country, 25 of 27 districts showed a substantial decline ranging from a 3.3% reduction to 79% reduction. By 2017, 16% of Malawi’s population still lived in areas that support *Pf*PR _2-10_ ≥ 25%.

**Conclusions: **Malawi has made substantial progress in reducing the prevalence of malaria over the last seven years. However, Malawi remains in *meso*-endemic malaria transmission risk. To sustain the gains made and continue reducing the transmission further, universal control interventions need to be maintained at a national level.

## Introduction

Malaria-endemic countries are increasingly encouraged to define their sub-national epidemiology to understand the likely rational allocation of intervention mixes and the changing malaria landscape [ WHO, 2010; WHO, 2015]. Within a country, variations in vector ecology, environment, and intervention coverage all determine the patterns of malaria risk and incipient disease burden. Consequently, maps of malaria risk are required by national malaria control programmes to guide decision making in heterogenous settings.

National cartographies of malaria risk were common during the 1950s and 1960s [ Snow & Noor, 2015]. Often these maps were based on accepted definitions of malaria endemicity [ Lysenko & Semashko, 1968; Metselaar & van Thiel, 1959], derived from community-based surveys of malaria infection prevalence and climate ecologies [ Snow & Noor, 2015]. The importance of malaria risk maps began to re-emerge during the 1990s, coincident with the pan-African malaria resurgent epidemic [ Snow *et al.*, 1996], resulting in a new generation of national malaria strategic plans that articulated the sub-national disparities in malaria ecology, transmission and disease burden [ Omumbo *et al.*, 2013].

In Malawi, as part of the 2001–2005 national malaria strategic plan [ NMCP, 2001], the epidemiology of malaria was described based on a World Health Organization (WHO) mission to the country 30 years earlier [ Cheyabejara *et al.*, 1974] “ *In 1973, the WHO mission determined malaria to be meso- to hyper- endemic in Malawi, except in isolated higher altitudes mountainous regions*” [ NMCP, 2001]. However, during the second national malaria strategic plan 2005–2010 [ NMCP, 2005], no references to the epidemiological patterns of transmission, dominant vector species or disease burden were provided.

It was not until 2006 that empirical data on malaria infection prevalence was used with model-based geostatistical (MBG) methods to provide predictive quantities of risk across Malawi [ Kazembe *et al.*, 2006]. This work used data on malaria prevalence from 73 survey locations, where children aged 1–10 years that had been sampled between 1970 and 2001. Temperature, rainfall, potential evapotranspiration, and elevation were all used as covariates to help predict infection prevalence at un-sampled locations using information and correlates with sampled locations. This map was used in the malaria programme review in 2010 [ NMCP, 2010] and the national strategic plan 2011–2015 to highlight the hyper-endemic nature of malaria transmission in the country, with variations in higher altitude areas [ NMCP, 2011].

In 2013, the application of MBG methods was extended to include 1057 surveys of malaria infection undertaken between 2000 to 2010, employing covariates related to urbanization and temperature suitability for malaria transmission to provide a 1×1 km posterior prediction of malaria risk in 2000, 2005 and 2010 [ Bennet *et al.*, 2013]. These analyses showed significant sub-national variations in malaria prevalence; however, there was little change across the prediction time-periods.

Since 2010, there have been significant additional community-based malaria surveys undertaken, sampled both at district and national levels. This paper describes the re-assembly of malaria infection data in Malawi and their use within a MBG framework to understand changing sub-national malaria endemicity between 2010 and 2017 at a time of increased malaria intervention coverage throughout the national strategic plan 2011–2015 [ NMCP, 2011].

## Methods

### Geography

**Figure 1.**
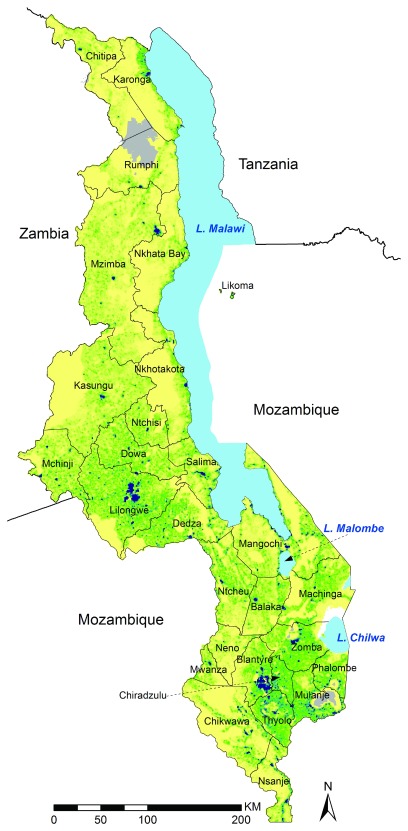
The Geography, population density, districts and unsuitability for malaria transmission in Malawi. Population density ranges from zero (yellow) to 37,332 person per 1 km grid (dark blue). Grey areas represent a temperature suitability index (TSI) of zero which indicates a temperature range that cannot support malaria parasite development cycles in the mosquito [ Gething *et al.*, 2011], and all correspond to unpopulated areas in the Nyika Plateau in the north and Mulange Massif range in the south.

Malawi is a landlocked country located in the South Eastern region of Africa along the Great Rift Valley, bordered by Tanzania, Mozambique and Zambia ( Figure 1). The country has an estimated population of 17.6 million people in 2017 [ NSO, 2018], and is one of the poorest countries in Africa with a per capita GDP of US$350 [ World Bank, 2018]. Altitude varies considerably from 37 metres above mean sea level (MASL) to 3003 MASL. Both proximity to Lake Malawi and altitude define the variable climate patterns [ Vincent *et al.*, 2014]. The country is divided into three regions, namely Northern, Central, and Southern regions, which encompass 28 districts ( Figure 1). This includes the small islands of Likoma and Chizumulu that form Likoma district, with *circa* 9,000 people, in Mozambique waters ( Figure 1). For the purposes of the present analysis Likoma district is excluded as it is situated more than 69 km from the mainland.

### Malaria control 20 years on

Malawi launched a national malaria strategic plan in 2001 in line with recommendations made by the Roll Back Malaria initiative [ NMCP, 2001]. With rapidly emerging sulphadoxine-pyrimethamine (SP) resistance, Malawi proposed a change in its first-line treatment policy from SP to Artemether-Lumefantrine (AL) in 2004, but the policy was not implemented until 2007–2008 [ Malenga *et al.*, 2009]. A programme of socially marketed, subsidized insecticide-treated net (ITN) distributions was launched in 1998 [ Mathanga *et al.*, 2012; MoH, 2005], and was largely the sole source of ITN access nationwide until 2007 when the free delivery of ITN through public health facilities to children attending immunization and pregnant mothers was launched [ Mathanga *et al.*, 2012]. Between 2007 and 2010, approximately 4 million nets were distributed free of charge to vulnerable populations, including 1.1 million nets provided during the first nationwide mass-campaign in 2008. Before 2010, only pilot indoor-residual house spraying (IRS) using pyrethroids were undertaken in small community studies in Ntchisi, Mzimba, Kayelekera uranium mine and the Nchalo and Dwangwa Estates of the Illovo sugar company [ Chunga & Kumwenda, 2014; MoH, 2011]. Between 2000 and 2010, partial increases in vector control coverage [ Chanda *et al.*, 2016] and the delayed introduction of effective therapeutics [ Malenga *et al.*, 2009] was reflected in limited reductions in national community-based malaria prevalence, 2000 (36.4%) and 2010 (36.3%) [ Bennett *et al.*, 2013] and increases over the same period in malaria hospitalizations [ Okiro *et al.*, 2013; Roca-Feltrer *et al.*, 2012a].

In 2011, the National Malaria Strategy 2011–2015 was launched with a vision that all people in Malawi are free from the burden of malaria and an ambition to halve the malaria disease and mortality burden by 2016 [ NMCP, 2011]. Substantial increases in long-lasting insecticide-treated net (LLIN) distribution were achieved from 2010 with over 1 million nets being delivered through public health facilities each year since 2011, and 5.4, 7.0 and 8.6 million nets distributed during a mass campaigns in 2012, 2014 and 2016, respectively. Between July 2010 and 2011, IRS using pyrethroids was undertaken in seven districts (Nkhotakota, Salima, Karonga, Nkhata Bay, Mangochi, Chikwakwa and Nsanje) protecting approximately 3 million people [ NMCP, 2012]. Fuel shortages, funding interruptions and increasing Lambda-cyhalothrin and carbamate resistance [ Mzilahowa *et al.*, 2016; Wondji *et al.*, 2012] led to a significant reduction in IRS activities, with Salima district only under IRS in 2013 where after IRS was suspended nationwide [ Chanda *et al.*, 2015]. In 2011, the introduction of rapid diagnostic tests (RDTs) was launched nationwide with supplies to peripheral health facilities and training of health workers in 2012 and 2013 [ PMI, 2016], which were included for health workers in hard to reach areas as part of integrated community case management (iCCM) in 2015 [ Phiri *et al.*, 2016].

### Malaria prevalence survey data assembly

The process of identifying community-based malaria survey data is described in detail elsewhere [ Bennett *et al.*, 2013; Snow *et al.*, 2017]. Of importance have been national household sample surveys that have included malaria infection prevalence among children in national micro-nutrient surveys conducted in 2001, 2006, 2009 and 2016; national demographic and/or malaria indicator surveys undertaken in 2010, 2012, 2014 and 2017 [ NMCP, 2017]; sub-national surveys undertaken at district levels by the College of Medicine Malaria Alert Centre between 2005–2009 [ Mathanga *et al.*, 2010], repeat surveys in three districts between 2012 and 2014 [ Walldorf *et al.*, 2015], and district-wide surveys in Chikwawa 2010–2016 [ Buchwald *et al.*, 2016; Kabaghe *et al.*, 2017; Kabaghe *et al.*, 2018a; McCann *et al.*, 2017; Roca-Feltrer *et al.*, 2012b]. Other data were derived from published sources and the generous help with unpublished data from national malaria scientists and collaborators, listed at the end of the paper. Data were restricted to surveys undertaken between January 2000 and December 2017.

For each of the identified data sources, information on the month and year of the survey, age range of the surveyed population, the numbers tested versus numbers identified as harbouring *Plasmodium falciparum* and the methods of parasite detection were all extracted. The location (longitude and latitude) of each surveyed village, school or enumeration cluster was checked using national statistical office high-resolution global positioning system (GPS) databases and other publicly available, online digital gazetteers. The raw information is provided as underlying data [ Chipeta *et al*., 2019; Snow, 2017].

### Geostatistical spatio-temporal analysis

MBG [ Diggle *et al.*, 1998; Diggle & Giorgi, 2019] is a likelihood-based approach that allows prediction of a health outcome of interest using sparsely sampled data. This modelling framework has also been extended to interpolate both the spatial and temporal variation of disease prevalence through the analysis of repeated cross-sectional data [ Giorgi *et al.*, 2018]. MBG has become a well-established tool in statistics for modelling the spatio-temporal correlation induced by unmeasured risk factors to predict prevalence at any desired place and time.

To model changes in *Pf*PR _2 *−*10_ by borrowing strength of information across time and space, an MBG model was used. Unlike previous MBG approaches [ Bennett *et al.*, 2013] a decision was made not to include human settlement, climate or other environmental covariates during the modelling exercise. The inclusion of covariates (climate, land use, social economic status and intervention), when used to assist predictions at locations without data, presume a clearly defined *a priori* biological relationship with prevalence and are only valuable when predictions must be made without large volumes of input empirical prevalence data, which themselves represent the product of all the possible covariate influences [ Macharia *et al.*, 2018].

The model is described as follows. Let *x* be the location of a surveyed community in year *t*. Define a spatio-temporal Gaussian process, *S*( *x, t*), and unstructured random effects, *Z*( *x, t*), to account for the unexplained variation between and within communities, respectively. Conditionally on *S*( *x, t*) and *Z*( *x, t*), the counts of positive tests for *P. falciparum* were assumed to follow mutually independent binomial distributions with number of trials *N*, corresponding to number of sampled individuals, and probability of a positive outcome *p*( *x, t*) at location *x* ( *n*=surveyed locations) and year *t* (2000–2017) given by

$$\log\left\{\frac{p(x,t)}{1-p(x,t)}\right\}=\alpha +\beta mA+\gamma MA+S(x,t)+Z(x,t).\;(1)$$

where *mA* and *MA* are the minimum and maximum age among the sampled individuals at a location *x* and time *t*. In carrying the spatio-temporal predictions, *mA* and *MA* were set to 2 and 10 respectively to standardise to the age group 2–10 years. A stationary and isotropic Gaussian process for the spatio-temporal random effects is assumed *S*( *x, t*), with an exponential correlation function given as

$$\;cor\left\{S(x,t),S(x^{\prime },t^{\prime })\right\}=e^{\left\{-\left\|u\right\|/\varphi \right\}}e^{\left\{-|v|/\psi \right\}}\;(2)$$

where *φ* and *ψ* are scale parameters which regulate the rate of decay of the spatial and temporal correlation for the increasing distance and time separation, respectively; *u* = || *x* − *x*′|| is the distance in space between the location of any two communities, one at *x* and the other at *x*′; *ν* = | *t* − *t*′| is the time separation in years between any two surveys.

The model parameters were estimated via Monte Carlo maximum likelihood in the R statistical software environment [ R Core Team, 2017] using the PrevMap package version 1.4.2 [ Giorgi & Diggle, 2017]. The targets for predictions were *Pf*PR _2−10_ over the 1×1 km regular grid covering the whole of mainland Malawi. Maps of malaria risk were generated for the two reference years 2010 and 2017 using ArcMap 10.4 (ESRI Inc., Redlands, CA, USA).

### Model validation

The model was validated using two methods. First by testing evidence against the residual spatio-temporal correlation in the data through the following variogram-based validation algorithm [ Giorgi *et al.*, 2018]: 1) Generate a point estimate *Z*( *x _i_*, *t _i_*) i.e. $\tilde{Z}(x_{i},t_{i})$ from a non-spatio-temporal model, for each observed location *x _i_* and time *t _i_*; 2) Permute the order of the data, including $\tilde{Z}(x_{i},t_{i})$, while holding ( *x _i_*, *t _i_*) fixed; 3) Compute the empirical semi-variogram for $\tilde{Z}(x_{i},t_{i})$; 4) Repeat steps (1) and (2) a large number of times, say *B;* 4) Using the resulting *B* empirical variograms to generate 95% confidence intervals at each of the pre-defined distance bins. To conclude that there is no evidence against the adopted spatio-temporal model correlation the empirical semi-variogram from the original data must fall within the generated 95% confidence intervals. Second, validation statistics based on a 10% hold-out dataset or correlation against observed and predicted estimates of *Pf*PR _2-10_, bias and mean absolute error was done.

### Population-adjusted risk

Neither human settlement nor malaria risk are evenly distributed, and therefore to ensure that malaria risk maps converge with human population density, similar gridded surfaces of population are required. Dasymetric modelling techniques for the reallocation of populations within census units have been developed to overcome the difficulties caused by input census data of coarse spatial resolution [ Linard *et al.*, 2012; Stevens *et al.*, 2015]. In brief, the 2008 Malawi national census, organized by 12,666 enumeration areas (lowest available level of aggregation), was reallocated using a Random Forest model in combination with land cover specific weightings, protected areas, night-time lights, roads, rivers, altitude and settlement data to adjust and re-allocate population densities within each enumeration area ( available here). United Nations rural and urban growth rates were used to project population’s forward to 2010 and 2017 ( available here) to provide a gridded dataset of population distribution (counts) at 0.1×0.1 km resolution. The population maps were resampled to 1×1 km grids ( Figure 1) to match the malaria risk mapped outputs.

## Results

### Description of survey data

**Figure 2.**
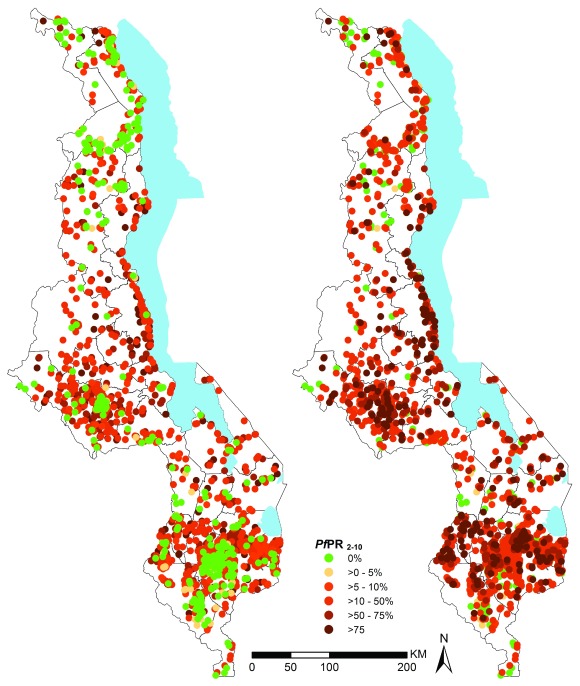
Spatial distribution of *Pf*PR _2-10_ surveys in Malawi between 2000 and 2017. Data assembled from 2,237 surveys at 1,834 unique locations of community parasite prevalence showing the lowest values of *Pf*PR _2-10_ on top (left panel) and highest values of *Pf*PR _2-10_ on top (right panel) to reflect locations sampled more than once during the period.

A total of 2,276 independent survey data points at 1,874 unique locations were identified during the data assembly process. Two survey locations could not be geo-coded and 37 surveys were undertaken on the islands of Likoma district and were excluded. Of the remaining 2,237 surveys, 403 (18%) were repeat surveys, taken at the same geo-location but in different years and 1,834 were unique locations. The data covered the entire period 2000–2017 and represented the examination of 59,920 individuals. The majority of surveys employed microscopy (83%), rather than RDTs (17%) for parasite detection. Most survey locations (86%) were positioned using local GPS sources. The location of the age-corrected survey data is shown in Figure 2.

### Spatio-temporal variation in malaria risk

**Figure 3.**
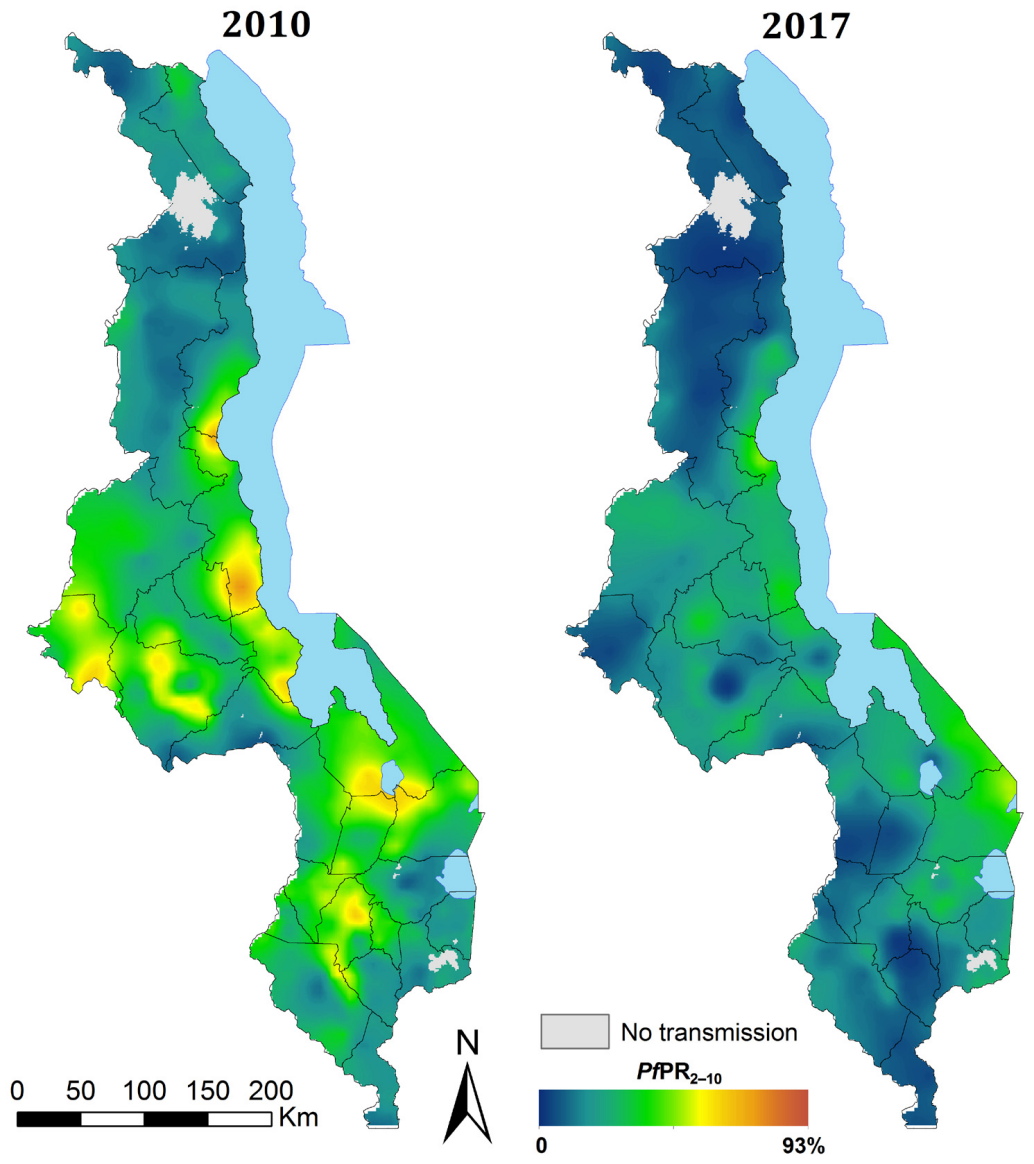
Mean standardized *Plasmodium falciparum* parasite rate ( *Pf*PR _2–10_) for 2010 (left) and 2017 (right). The predicted posterior mean community *Pf*PR _2–10_ is presented at 1×1 km ranging from zero (dark blue) to 93% (dark red) in Malawi. Grey areas represent TSI values of zero, unable to support transmission.

**Figure 4.**
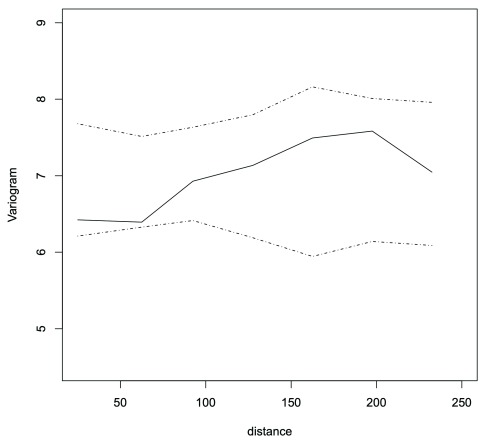
Validity of the assumed covariance model for the spatial correlation. The empirical semi-variogram (solid line) falls within the 95% tolerance intervals (dashed lines), indicating that the adopted covariance model was compatible with the data.

The assembled data were used in the spatio-temporal model Equation 1 to generate the 1×1 km grids of mean predictions of *Pf*PR _2–10_ in 2010 and 2017 ( Figure 3). The validity of the spatio-temporal model indicated that the empirical semi-variogram falls within the 95% confidence intervals ( Figure 4), indicating that there is no evidence against the adopted spatio-temporal model. The predictive performance of the model using cross validation, holding 10% of the total sample (224 surveys), indicated a high correlation between observed and predicted *PfPR*_2 *−*10_ with *ρ* = 0.72, a bias of 0.28% and mean absolute error of 15%.

**Figure 5.**
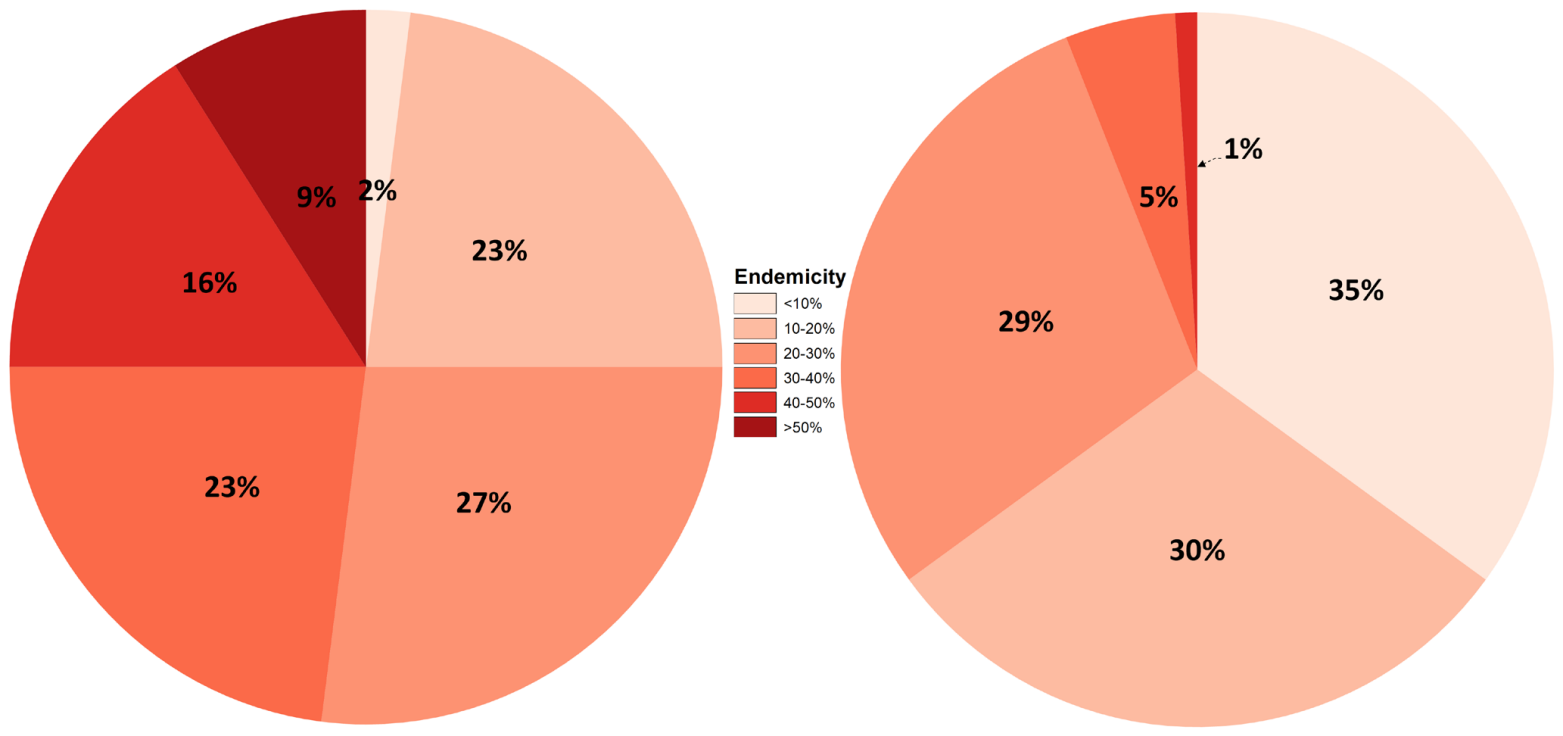
Percentage population of people living under different endemicity classes in 2010 (left) and 2017 (right). The mean community *Plasmodium falciparum* parasite rate ( *Pf*PR _2–10_) have been grouped into six classes ranging from light red (<10%) to dark red (>50%).

**Figure 6.**
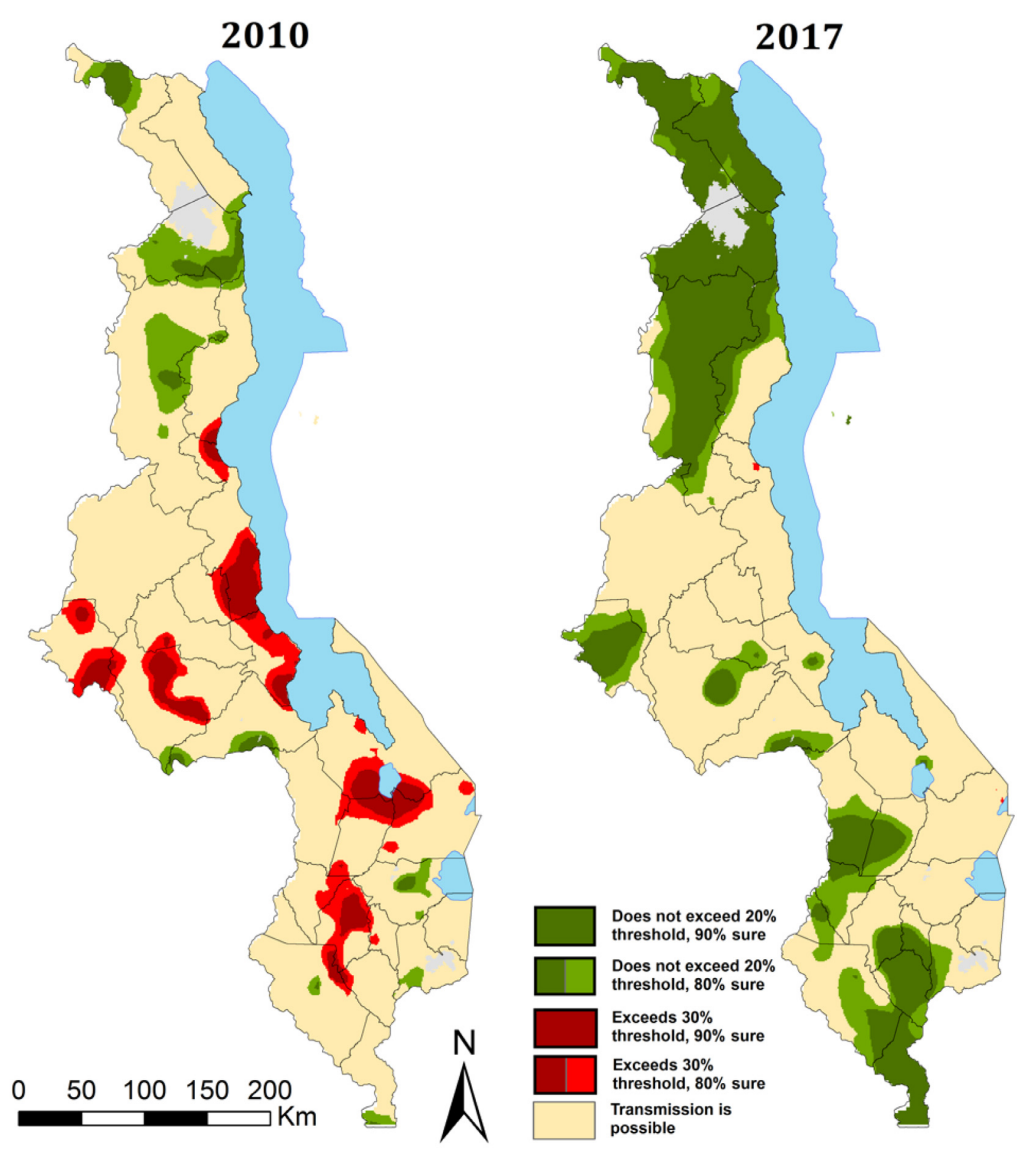
Non - exceedance and exceedance probabilities map. Showing areas where predicted P *f*PR _2–10_ is less (non-exceedance probability) than 20% which were > 80% confidently predicted (light green and dark green) or > 90% confidently predicted (dark green); and areas where P *f*PR _2–10_ is greater (exceedance probability) than 30% which were > 80% confidently predicted (light red and dark red) or > 90% confidently predicted (dark red). Areas which do not support malaria transmission are shown in grey (see Figure 1); all other areas where transmission can occur are shown in yellow.

**Figure 7.**
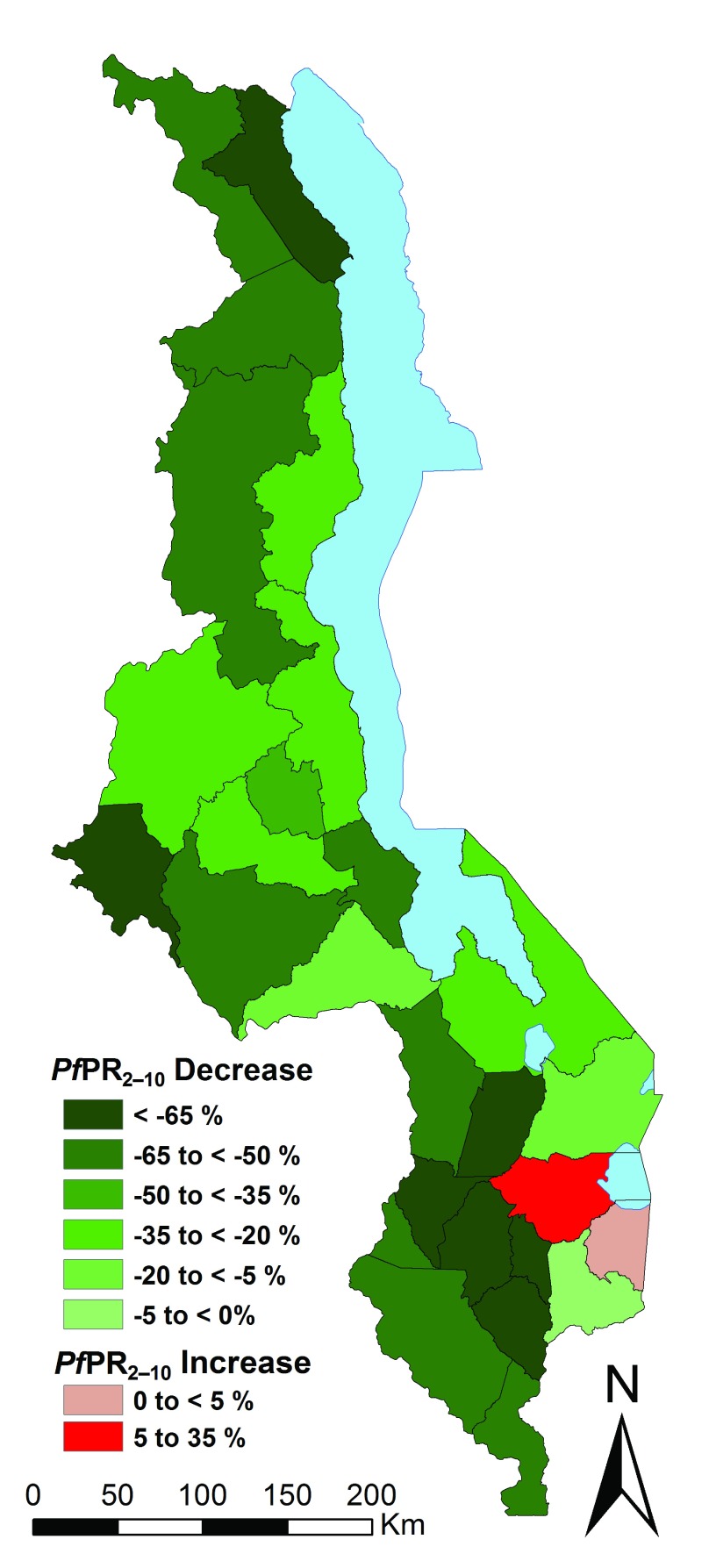
Percentage change in predicted mean *Pf*PR _2–10_ by district, between 2010 and 2017. The percentage change in mean *Pf*PR _2–10_ is shown in shades of green for decreasing malaria risk and shades of red for increasing risk.

The national mean predicted *Pf*PR _2–10_ in 2010 was 29.4% (95% confidence interval (CI) 26.6–32.3%) compared to 15.6% (95% CI 13.3–18.0%) in 2017. When combined with population density in each year this corresponds to a reduction of 94.7% in the numbers of people living in areas where *Pf*PR _2–10_ is greater than 40% and a corresponding 216.3% increase in populations living under areas of *Pf*PR _2–10_ <20% ( Figure 5). As shown in Figure 3 and Figure 6, declines in *Pf*PR _2-10_ were witnessed nationwide. However, the largest declines (≥60% reductions) in the population-weighted mean *Pf*PR _2-10_ by 2017 using 2010 as the baseline were observed in Karonga, Rumphi, Mchinji, Lilongwe, Balaka, Blantyre, Chiradzulu, Neno and Thyolo districts ( Table 1; Figure 7). Conversely, those witnessing the lowest reductions (<10%) by 2017 compared to 2010 were observed in Dedza and Mulanje districts and two districts showing a rise during the interval, Zomba and Phalombe districts ( Table 1; Figure 7). However, the confidence intervals for prevalence changes in Machinga, Mulanje and Phalombe districts contain a zero. To emphasise on prevalence decline, we categorised prevalence into two thresholds. In Figure 6, we show areas with low P *f*PR _2–10_ prevalence where prevalence lies below 20% (non-exceedance probability-NEP, 80 or 90% sure) and areas with very high prevalence where P *f*PR _2–10_ prevalence is above 30% (exceedance probability-EP, 80 or 90% sure), i.e. areas where intensive and sustained control is required.

**Table 1.** Predicted average *Pf*PR _2 *-*10_ population adjusted and relative change in 27 districts between 2010 and 2017.

District	*Pf*PR _2-10_ Population adjusted estimates, %			Number of surveys
	2010	2017	Per cent change (95% CI)	
Northern region *				
Chitipa	13.2	6.3	-52.7 (-59.7, -46.6)	29
Karonga	19.1	5.3	-72.1 (-74.9, -68.2)	94
Mzimba	15.1	7.1	-53.1 (-53.5, -50.8)	109
Nkhata Bay	30.7	20.3	-33.9 (-21.1, -41.3)	28
Rumphi	9.5	3.5	-63.1 (-59.8, -64.0)	176
Central region				
Dedza	20.0	18.2	-8.4(-2.5, -9.2)	45
Dowa	30.6	20.5	-33.2(-38.1, -27.5)	26
Kasungu	27.0	19.0	-29.6 (-40.6, -20.0)	35
Lilongwe	36.2	13.8	-61.9 (-69.7, -54.6)	266
Mchinji	49.4	10.4	-79.0 (-86.5, -68.8)	19
Nkhotakota	48.2	31.7	-34.3 (-48.3, -20.9)	87
Ntcheu	31.9	14.1	-55.8 (-63.2, -48.4)	22
Ntchisi	36.6	23.7	-35.2 (-47.5, -26.8)	11
Salima	48.0	19.5	-59.5 (-70.9, -48.5)	31
Southern region				
Balaka	39.9	12.8	-68.0 (-76.3, -58.5)	24
Blantyre	36.0	7.9	-78.2 (-85.2, -70.9)	277
Chikwawa	26.9	12.2	-54.8 (-71.0, -38.7)	205
Chiradzulu	33.3	11.4	-65.9 (-72.8, -59.1)	150
Machinga	35.2	31.4	-10.6 (-34.5, 7.9)	32
Mangochi	40.8	27.0	-33.8 (-43.4, -26.1)	52
Mulanje	18.2	17.6	-3.3 (-16.0, 4.9)	27
Mwanza	29.0	11.7	-59.8 (-70.0, -51.7)	51
Neno	39.9	11.3	-71.6 (-83.8, -55.6)	72
Nsanje	15.6	6.7	-56.8 (-68.7, -48.0)	15
Phalombe	19.5	19.6	0.26 (-12.2, 8.0)	158
Thyolo	19.5	6.8	-65.2 (-77.8, -52.7)	69
Zomba	18.3	24.7	34.9 (20.7, 47.2)	90
Total	**29.4**	**15.6**	**-47.2 (-50.2, -44.4)**	

*Predictions do not include Likoma Island, which is in northern Lake Malawi.

## Discussion

Earlier investigations of changing malaria prevalence [ Bennett *et al.*, 2013] and malaria hospitalisation [ Okiro *et al.*, 2013; Roca-Feltrer *et al.*, 2012a] suggested that between 2000 and 2010 there was little evidence in support of a reduction in malaria transmission or disease burden. The launch of the 2011, five-year national malaria strategic plan, over US$150 million in donor support has been provided for malaria control between 2010–2015 [ WMR, 2018], resulting in a significant increase in vector control coverage and improved malaria case-management, including expanded community-based care. This has corresponded with a dramatic decline in infection prevalence nationwide. Compared to 2010, the national mean *Pf*PR _2-10_ has declined by 47.2% ( Figure 3, Figure 6 and Figure 7; Table 1). In 2017, 6% of the population lived under conditions of intense malaria transmission ( *Pf*PR _2-10_ >40%) compared to 25% in 2010 ( Figure 5). It is not possible to directly attribute the reduction in malaria transmission to any specific intervention or combination of interventions. However, in the absence of any significant climate anomalies during this interval that might have lowered malaria risks [ Future Climate for Africa, 2017], it seems plausible that the reductions seen were a result of direct intervention.

It should, however, be highlighted that Malawi remains a highly malaria endemic country. Over 16.4% of the population live in areas where at least 1 in 4 children aged 2–10 years harbour malaria infection; the populations living in districts along the shore of Lake Malawi and central region remain under highest risk. Notably, there are few areas where extremely low, pre-elimination conditions exist ( *Pf*PR _2-10_ <1%) [ Cohen *et al.*, 2010]. Consequently, the National Malaria Control Programme (NMCP) needs to sustain efforts at universal infection prevention and disease management. Unlike neighbouring countries, which exhibit a substantial sub-national diversity of malaria transmission warranting a sub-national tailoring of interventions, Malawi should be considered as a country that should, at present, maintain a single, national strategic approach to malaria control.

The present analysis focuses on malaria infection prevalence, a frequently used malaria metric and used for over 100 years across Africa [ Snow *et al.*, 2017]. Prevalence data for Malawi have been assembled from multiple sources including district-level research platforms, school surveys and nutritional surveys. The leveraging of multiple national survey data from diverse research and health constituents improves the precision of predictions over sparse data collected during single cross-sectional national malaria or health surveys powered to provide information on variables other than prevalence at low spatial resolutions. Malawi has several sentinel research districts which provide platforms to investigate specific intervention access, attribution and impact questions [ Buchwald *et al.*, 2016; Escamilla *et al.*, 2017; Kabaghe *et al.*, 2018a; Kabaghe *et al.*, 2018b; Roca-Feltrer *et al.*, 2012a]; these serial, repeat observational data significantly contribute to informing the changing national profile of malaria risk in the MBG models. A key function of the NMCP remains curating and updating these data from all partners in-country. However, the analysis of sub-national variations in risk and epidemiological transitions should be triangulated with additional routine data from health information systems and malaria hospitalization. Through a process of data triangulation, a more granular understanding of the epidemiological transition is possible. The use of MBG methods to interpolate information on malaria prevalence at community levels, is less perfect when compared to complete, reliable routine data on the monthly presentation of parasitologically diagnosed fevers to health facilities. However, in the absence of complete routine data, both routine and survey data provide opportunities to understand the impact of scaled intervention on the malaria burden sub-nationally.

## Conclusion

Malawi has made substantial progress in reducing the prevalence of malaria over the last seven years. It seems plausible that this transition has been a direct result of substantial investment in improving the scale and range of intervention coverage. More detailed interrogation, and triangulation, of intervention and routine data, is required to understand the sub-national impact of control. Malawi remains a high burden country. To accelerate future progress will require further prioritization of existing interventions and increasing their reach for several years before sub-national targeting of resources becomes a priority.

## Data availability

Figshare: Geostatistical analysis of Malawi’s changing malaria transmission from 2010 to 2017. https://doi.org/10.6084/m9.figshare.7856990.v2 ( Chipeta *et al.*, 2019).

This project contains survey data from 2000–2017.

Data are available under the terms of the Creative Commons Attribution 4.0 International license (CC-BY 4.0).
