# Supplementary material for: Geostatistical analysis of Malawi’s changing malaria transmission from 2010 to 2017
Source: Wellcome Open Res. 2019 Jul 4;4:57. Originally published 2019 Mar 27. [Version 2] doi: 10.12688/wellcomeopenres.15193.2 (PMC6662685; doi:10.12688/wellcomeopenres.15193.2)
Supplement: Peer review history

# Peer review: Reviewer response for version 2

**DOI:** 10.21956/wellcomeopenres.16757.r35914

**Published:** 2019-07-29

**Authors:** Theresa Smith

**Affiliations:** 1 Department of Mathematical Sciences, University of Bath, Bath, UK

The authors have addressed my concerns.

I confirm that I have read this submission and believe that I have an appropriate level of expertise to confirm that it is of an acceptable scientific standard.

# Peer review: Reviewer response for version 2

**DOI:** 10.21956/wellcomeopenres.16757.r35913

**Published:** 2019-07-26

**Authors:** Rocco Panciera

**Affiliations:** 1 United Nation Children’s Fund (UNICEF), New York, NY, USA

The authors have addressed my concerns satisfactorily.

I confirm that I have read this submission and believe that I have an appropriate level of expertise to confirm that it is of an acceptable scientific standard.

# Peer review: Reviewer response for version 1

**DOI:** 10.21956/wellcomeopenres.16576.r35579

**Published:** 2019-06-06

**Authors:** Theresa Smith

**Affiliations:** 1 Department of Mathematical Sciences, University of Bath, Bath, UK

In this paper, Chipeta and co-authors use model based geostatistics to analyse malaria prevalence data collected in a large number of surveys throughout Malawi and across several years. There is a particular emphasis on estimating changes in prevalence from 2010 to 2017 during which several malaria control strategies were implemented. Using a spatio-temporal model taking into account geographic and time correlations between surveys as well as unstructured heterogeneity, the authors produce estimates of *Plasmodium falciparum* prevalence in children aged 2-10 (PfPR_2-10) at a spatial resolution of 1km x 1km in 2010 to 2017 along with regional estimates in the change in prevalence for 27 districts.

 Overall, this is a well-written, technically sound paper. I have some small questions and suggestions:

**Are sufficient details of methods and analysis provided to allow replication by others?/**

**Are all the source data underlying the results available to ensure full reproducibility?**

 I could not find the source data in the links the authors provided. For example, the figshare reference (Chipeta *et al.*, 2019) does not contain the minimum and maximum age or the month of the survey.

 Could the authors provide R code for their models and data preparation? In particular, the population re-adjustment and age standardisation are not described in enough details to allow replication by others.

**Are the conclusions drawn adequately supported by the results?**

 Some of the conclusions in the discussion do not seem fully supported by the statistical results presented. The authors state, on page 9, ‘the populations living in districts along the shore of Lake Malawi and central region remain under highest risk.’ It seems to me (a statistician with limited expertise in the application) that Phalombe and Zomba would be at highest risk because these are the two regions with potentially increasing prevalence (especially Zomba). Perhaps the authors could elaborate on how they determined ‘highest risk’?

 On the same page, the authors recommend, ‘Malawi should be considered as a country that should, at present, maintain a single,national strategic approach to malaria control’. How is this recommendation supported by the modelling?

**Some smaller questions**

 Are the underlying survey data based on random samples from each location? Are there potential design factors or biases that should to be taken into account (as in Giorgi *et al*., 2014 ^1^)?

 Is month used in the temporal part of the model or year of the survey used? Only year is in the shared data.

 Fig 3: How much uncertainty is there in these estimates?

 Fig 5: How much uncertainty is there in the classification? A different figure type (perhaps a stacked bar chart) would help emphasize the decrease in prevalence between the two years.

 Table 1: Could the authors add the number of surveys per district as a column in this table?

I confirm that I have read this submission and believe that I have an appropriate level of expertise to confirm that it is of an acceptable scientific standard, however I have significant reservations, as outlined above.

## References

[1] : Combining data from multiple spatially referenced prevalence surveys using generalized linear geostatistical models. *Journal of the Royal Statistical Society: Series A (Statistics in Society)*.2015;178(2) : 10.1111/rssa.12069 445-464 10.1111/rssa.12069

# Response

**Published:** 2019-06-19

**Authors:** Michael Give Chipeta

**Affiliations:** Malawi Liverpool Wellcome Trust, Malawi

We thank Dr Theresa Smith for thoughtful and helpful comments made on the first version.  We have improved the clarity of the paper by responding to the points raised as follows: 

**Summary**

In this paper, Chipeta and co-authors use model based geostatistics to analyse malaria prevalence data collected in a large number of surveys throughout Malawi and across several years. There is a particular emphasis on estimating changes in prevalence from 2010 to 2017 during which several malaria control strategies were implemented. Using a spatio-temporal model taking into account geographic and time correlations between surveys as well as unstructured heterogeneity, the authors produce estimates of *Plasmodium falciparum *prevalence in children aged 2-10 (PfPR_2-10) at a spatial resolution of 1km x 1km in 2010 to 2017 along with regional estimates in the change in prevalence for 27 districts.

Overall, this is a well-written, technically sound paper. I have some small questions and suggestions:

1. I could not find the source data in the links the authors provided. For example, the figshare reference (Chipeta *et al.*, 2019) does not contain the minimum and maximum age or the month of the survey.

**Response: **The data contains the year of the survey only as described in the data section of the manuscript. We have now updated the dataset on figshare repository ( here), that contains minimum and maximum ages **. **We have also added this link in the updated manuscript.

2. Could the authors provide R code for their models and data preparation? In particular, the population re-adjustment and age standardisation are not described in enough details to allow replication by others.

**Response: **The assembled surveys contained a variety of sampled age groups and it is necessary to have a single age-group in comparing data in time and space. The theory behind changing age profiles of infection prevalence depending on the intensity of transmission in a given location is provided in the reference by Smith *et al*. (2007). To standardise age to a single age range of 2 – 10 years (P *f*PR _2-10_), we incorporate this process in the model by setting minimum ( *mA)* and maximum ( *MA)* ages to 2 and 10 respectively, in Equation (1) in the manuscript. Analysis was carried out in PrevMap package in R, the code is available upon reasonable request to the first author (MGC) (this is being prepared for online hosting). Population adjustment (to account for where people live) was done in ArcMap 10.4, using WorldPop data. We provide the details in the manuscript on the links available here and here. 

Smith DL, Guerra CA, Snow RW, Hay SI. Standardizing estimates of the Plasmodium falciparum parasite rate. Malar J. 2007; 6:131.

3. Some of the conclusions in the discussion do not seem fully supported by the statistical results presented. The authors state, on page 9, ‘the populations living in districts along the shore of Lake Malawi and central region remain under highest risk.’ It seems to me (a statistician with limited expertise in the application) that Phalombe and Zomba would be at highest risk because these are the two regions with potentially increasing prevalence (especially Zomba). Perhaps the authors could elaborate on how they determined ‘highest risk’?

**Response: **Highest risk is a factor of the change between the two timepoints and the current risk (in 2017) We agree with the reviewer, Phalombe and Zomba districts have the high prevalence, in addition, the transmission risk along the lake shore and central region remains high because of the enabling environment for the vectors. At the same time, the districts along the lake shore and central region have experienced least changes despite the interventions and control efforts.

4. On the same page, the authors recommend, ‘Malawi should be considered as a country that should, at present, maintain a single, national strategic approach to malaria control’. How is this recommendation supported by the modelling?

**Response: **This is based on WHO recommendations on subnational stratification for malaria control **. **Malawi remains a meso-endemic country, with very few with extremely low pre-elimination settings (< 1% of the population). This in our view, still calls for universal coverage control effort. Targeted interventions should be applied in pre-elimination and highly heterogeneous areas with substantial areas covering high and others covering low risk.

5. Are the underlying survey data based on random samples from each location? Are there potential design factors or biases that should to be taken into account (as in Giorgi *et al*., 2014 ^1^)?

**Response:**  The underlying surveys are based on a collection of multiple random surveys. For national household surveys, these are provided in the links to national household survey designs, mostly two-stage random surveys. For other published works, these are often provided in detail in the peer reviewed publications. Overall, our only requirement was that a community or a school was sampled as a single cross-section and that information was available on the date, number examined and numbers positive. It is beyond the scope of these large data assembly approaches, levering all possible national survey data, to build a detailed sampling strategy per data point. This is possible when ONLY using multi-stage, household sample surveys. And some investigators only use these for mapping work, building in survey weights. However, it is our view that for NMCPs, these are often limited in the information they can provide on malaria prevalence and ignore the vast amounts of other unpublished works that exist within a country. Here we have levered all possible data from all possible sources to be able to provide the most complete source of data on malaria infection prevalence in Malawi.

6. Is month used in the temporal part of the model or year of the survey used? Only year is in the shared data.

**Response:** Only year of the survey is used in the modelling of the data.

7. Fig 3: How much uncertainty is there in these estimates?

**Response: **Uncertainty in the estimates was quantified using the standard errors. The standard errors ranged from 0.0005 to 0.3 across space and time and were largely influenced with availability of data across space and time. Areas with more data were seen to have low standard errors and narrow Confidence Intervals. Standard error maps are available upon request to the first author (MGC).

8. Fig 5: How much uncertainty is there in the classification? A different figure type (perhaps a stacked bar chart) would help emphasize the decrease in prevalence between the two years.

**Response: **To emphasise the decrease in prevalence between 2010 and 2017, we now provide a new Figure for exceedance and non-exceedance probabilities maps, showing areas where predicted P *f*PR _2–10 _is less (non-exceedance probability) than 20% which were > 80% confidently predicted or > 90% confidently predicted; and areas where P *f*PR _2–10_ is greater (exceedance probability) than 30% which were > 80% confidently predicted or > 90% confidently predicted, for both 2010 and 2017.

9. Table 1: Could the authors add the number of surveys per district as a column in this table?

**Response: **We have added this information in Table 1 as suggested.

Once again, we thank the reviewer and offer the improved manuscript based on this and two other reviews.

# Peer review: Reviewer response for version 1

**DOI:** 10.21956/wellcomeopenres.16576.r35159

**Published:** 2019-05-21

**Authors:** Tobias Chirwa

**Affiliations:** 1 Division of Epidemiology and Biostatistics, School of Public Health, University of the Witwatersrand, Johannesburg, South Africa

**Summary**

 I have read the manuscript report and, subject to addressing my comments, should be considered for indexing. The paper looks at prevalence of malaria in space and time, taking into account multiple data sources to counter sparse data. The setting is Malawi and the authors utilize various data sources to estimate prevalence in 2010 and 2017. They argue that this change can be attributed to interventions implemented during the period. Generally, it is an important paper showing decline in prevalence over time.

 General comment:

 The authors need to justify why they only considered changes or trends based on two time points (i.e. 2010 and 2017). They had an opportunity to optimally use the different multiple sources over time. One would have assumed the estimation would be enriched with more time points considering surveys were also conducted in the intermediate period. Can the method provide estimates for several time points? For the two time points, I therefore feel the authors should be cautious of use of statistical significance.

 Specific comments:

 Abstract:

 Under results, it is not clear whether the 1834 communities are repeat communities and whether this was taken into account during analysis i.e. weighting.

 On declining prevalence, it is not clear why the authors say the decline was significant, and how this was determined. Was this by district, 2017 vs 2010?

 Introduction:

 In paragraph 5, the authors should clarify whether the 1057 surveys are in addition to the 73 mentioned in the previous paragraph.

 Method:

 The authors should give an exclusion and inclusion criteria. At the moment, it is not clear why Likoma District was excluded, although distance is mentioned.

 On page 4 – the authors show that there were different interventions in selected districts. Did these overlap and how were they handled in the analysis? Were the mass campaigns a national effort? And how do we explain the differences in prevalence decline?

 Under section on malaria prevalence the dates are from 2000 to 2017 while the title states 2010 to 2017 – this has been repeated elsewhere. Can the authors also explain how the age standardization was done?

 Under the geostatistical spatio-temporal analysis, can the authors explain the model and how this fits in with sparse data scenario considering national surveys were used? What would also be useful is to be able to compute inter-cross sectional survey estimates besides 2010 and 2017 since we have direct estimates at the time of cross-sectional surveys. I am looking for added value of the model than classical analysis.

 On Page 5 (population-adjusted risk), can the authors explain whether they adjusted for repeat surveys?

 Results:

 There are varying case detection methods from survey to survey. How were these used to estimate prevalence. Sensitivity, specificity issues? Under Table 1, I wish there were estimates for intermediate years. I think the authors have not optimally utilized the data. A trend analysis would be useful as well. Can the authors explain the small changes in some districts; and also why there is such a wide variation in the changes by district?

 Discussion:

 Can the authors discuss the design variation for the different multiple sources of data; One of the references has same title as the manuscript.

 Do we have any reference to climatic changes within the period of analysis?

I confirm that I have read this submission and believe that I have an appropriate level of expertise to confirm that it is of an acceptable scientific standard, however I have significant reservations, as outlined above.

# Response

**Published:** 2019-06-19

**Authors:** Michael Give Chipeta

**Affiliations:** Malawi Liverpool Wellcome Trust, Malawi

We thank Prof Tobias Chirwa for thoughtful and helpful comments made on the first version. We have improved the clarity of the paper by responding to the points raised as follows: 

**Summary**

I have read the manuscript report and, subject to addressing my comments, should be considered for indexing. The paper looks at prevalence of malaria in space and time, taking into account multiple data sources to counter sparse data. The setting is Malawi and the authors utilize various data sources to estimate prevalence in 2010 and 2017. They argue that this change can be attributed to interventions implemented during the period. Generally, it is an important paper showing decline in prevalence over time.

**A) General comment:**

The authors need to justify why they only considered changes or trends based on two-time points (i.e. 2010 and 2017). They had an opportunity to optimally use the different multiple sources over time. One would have assumed the estimation would be enriched with more time points considering surveys were also conducted in the intermediate period. Can the method provide estimates for several time points? For the two time points, I therefore feel the authors should be cautious of use of statistical significance. 

**Response:** We used all the available malaria survey data in Malawi, which included national surveys, subnational surveys, published and unpublished sources between 2000 and 2017 to make predictions to two-time points 2010 and 2017. Changes in prevalence between 2000 and 2010, using data assembled up to 2010, have been presented elsewhere by Bennet *et al*. (2013). The current work updated data through to 2017. Our aim was to explore changes in malaria prevalence in 2017 compared to 2010. It is always tempting to estimate malaria every year, but we argue that predictions should be made to years of maximal data, and years that are important for national malaria programme planning. We used data pre-2010 simply to improve the 2010 prediction.

We have taken note and revised accordingly to replace the word “significance” with “substantial” to better present our findings on the changes between estimates in 2010 and 2017. Additionally, we have added a map of exceedance and non-exceedance probabilities to quantify with 80% or 90% certainty that a location is above or below a given prevalence threshold.

Bennett A, Kazembe L, Mathanga DP, Kinyoki D, Ali D, Snow RW, Noor AM (2013). Mapping malaria transmission intensity in Malawi, 2000-2010. American Journal of Tropical Medicine & Hygiene, 89: 840–849

**B) Specific comments**: **Abstract:**

1. Under results, it is not clear whether the 1834 communities are repeat communities and whether this was taken into account during analysis i.e. weighting. 

**Response:** The 1834 are unique communities, among the 2237 communities. That’s 403 (18%) were repeat survey communities, taken at the same geo-location but in different years.

**Response:** Weighting was directly taken into account by the spatio-temporal correlation structure as defined in equation (2). More specifically, locations that are repeatedly sampled will have a spatial correlation of 1 (first factor on the right-hand side of equation (2)) but the model will still allow for variation over time as resulting from the temporal correlation (second factor on the right-hand side of the equation (2)) and from the unstructured random effects in equation (1).   

2. On declining prevalence, it is not clear why the authors say the decline was significant, and how this was determined. Was this by district, 2017 vs 2010?

**Response:** The prevalence decline was determined by the percentage change per district, from Table 1. We have reworded “significance” to “substantial”, indicating the change between prevalence in 2010 and 2017.

**Introduction:**

3. In paragraph 5, the authors should clarify whether the 1057 surveys are in addition to the 73 mentioned in the previous paragraph. 

**Response:** The 1057 surveys include the 73 surveys mentioned in the previous paragraph.

4. The authors should give an exclusion and inclusion criteria. At the moment, it is not clear why Likoma District was excluded, although distance is mentioned. 

**Response:** The model-based geostatistical methodology used for analysis in the current paper models prevalence by borrowing strength of information across space and time. Considering that the islands are at a distance of more than 60 kilometres from the mainland, it was deemed necessary to exclude them from the analysis. Spatial dependence of information tends to diminish with increasing separation distance and this island setting may have a different ecology, by its island status, to the contiguous areas of mainland Malawi.

5. On page 4 – the authors show that there were different interventions in selected districts. Did these overlap and how were they handled in the analysis? Were the mass campaigns a national effort? And how do we explain the differences in prevalence decline?

**Response:** Indeed, the interventions were overlapping, including insecticide treated-bed nets and indoor residual spraying. Net distribution campaigns were a nation-wide effort for years 2008, 2011, 2012, 2014 and 2016. IRS, however, was applied in a select number of districts as outlined in the paper. We intentionally did not include interventions as a variable in the analysis. The aim was to describe parasite prevalence without adjusting for, or testing for, interventions or other variables that might influence the prevalence of infection. We feel that climate, intervention, ecological changes would be reflected in the parasite rate in a given year. Therefore, the spatial predictions in 2010 and 2017 reflect the effects of intervention at these times. We have equally avoided specific statistical testing of intervention. Rather we have taken a plausibility approach [Habbict *et al*., 1999] and used in previous works looking at changing malaria infection prevalence in Africa [Snow *et al*., 2017; Macharia *et al*., 2018; Giorgi *et al*., 2018]. Whether the interventions were overlapping or not, their cumulative effect is reflected in the prevalence and changes in prevalence.

Giorgi E, Osman AA, Hassan AH, Ali AA, Ibrahim F, Amran JGH, et al. Using non-exceedance probabilities of—relevant malaria prevalence thresholds to identify areas of low transmission in Somalia. Malar J. 2018;17:88.

Habicht JP, Victora CG, Vaughan JP. Evaluation designs for adequacy, plausibility and probability of public health programme performance and impact. Int J Epidemiol. 1999;28:10–8.

Macharia PM, Giorgi E, Noor AM, Waqo E, Kiptui R, Okiro EA, Snow RW (2018). Spatio-temporal analysis of Plasmodium falciparum prevalence to understand the past and chart the future of malaria control in Kenya. Malaria Journal, 17: 340.

Snow RW, Sartorius B, Kyalo D, Maina J, Amratia P, Mundia CW, Bejon B, Noor AM (2017). The prevalence of Plasmodium falciparum in sub Saharan Africa since 1900. Nature, 550: 515-518.

6. Under section on malaria prevalence the dates are from 2000 to 2017 while the title states 2010 to 2017 – this has been repeated elsewhere. Can the authors also explain how the age standardization was done?

**Response:** The estimated prevalence was at two-time points (2010 and 2017). However, we used data pre-2010 (between 2000 and 2010) to provide stable estimates in 2010 by leveraging on the temporal autocorrelation.

**Response:** The assembled surveys contained a variety of sampled age groups and it is necessary to have a single age-group in comparing data in time and space. The theory behind changing age profiles of infection prevalence depending on the intensity of transmission in a given location is provided in the reference by Smith *et al*. (2007). To standardise age to a single age range of 2 – 10 years (P *f*PR _2-10_), we incorporate this process in the model by setting minimum ( *mA)* and maximum ( *MA)* ages to 2 and 10 respectively, in Equation (1).

Smith DL, Guerra CA, Snow RW, Hay SI. Standardizing estimates of the Plasmodium falciparum parasite rate. Malar J. 2007; 6:131.

7. Under the geostatistical spatio-temporal analysis, can the authors explain the model and how this fits in with sparse data scenario considering national surveys were used? What would also be useful is to be able to compute inter-cross sectional survey estimates besides 2010 and 2017 since we have direct estimates at the time of cross-sectional surveys. I am looking for added value of the model than classical analysis. 

**Response:** The model implemented is explained under this said section. A binomial model was implemented to model probability of having a positive outcome(infected) (Equation 1). The model had a stationary and isotropic Gaussian process, to account for the spatio-temporal random effects modelled with an exponential correlation function (equation 2 on the manuscript). Is the Gaussian noise/unexplained variation within communities or the unstructured random effects. The parameters were estimated using Monte Carlo maximum likelihood. All the data were considered as an individual survey point surveyed at a certain location and specific time irrespective of whether it was a cross section national survey or several data points from a village. Our rationale for selecting two time-periods is discussed earlier.

8. On Page 5 (population-adjusted risk), can the authors explain whether they adjusted for repeat surveys? 

**Response:** Population adjusted risk in this case means adjusting for uneven distribution of population when calculating malaria risk. The use of repeat surveys in the same location is discussed above.

**Results:**

9. There are varying case detection methods from survey to survey. How were these used to estimate prevalence. Sensitivity, specificity issues? 

**Response:** As detailed in the description of survey data: Most surveys employed microscopy (83%), rather than RDTs (17%) for parasite detection. The only gold standard is the PCR, however, they are too few to model and compare. Standardizing between microscopy and RDTs is fraught with difficulties because there are often very few details per survey, where both used, to describe the quality of microscopy (e.g. quality of staining, slide storage, how many high-power fields examined, and magnification of microscopy) nor any reliable algorithm to standardize between different RDTs. Where surveys have compared high quality slide reading and RDTs, for example in Kenyan school children, there has always been a close correlation between methods [Gitonga *et al*., 2012]. During previous work by the Malaria Atlas Project, supplementary information to data use described results obtained by RDTs and Microscopy as equivalent for the purposes of risk mapping [Gething *et al*., 2011]. For these reasons we did not attempt to standardize between detection methods. Future work, might provide novel ways of standardising between diagnostic methods, however at present these reliable algorithms are not available.

Gething PW, Patil AP, Smith DL, Guerra CA, Elyazar IR, Johnston GL, et al. A new world malaria map: Plasmodium falciparum endemicity in 2010. Malaria journal. 2011;10:378.  

Gitonga CW, Kihara JH, Njenga SM, Awundo K, Noor AM, Snow RW et al. Use of rapid diagnostic tests in malaria school surveys in Kenya: does under-performance matter for planning malaria control? Am J Trop Med Hyg. 2012; 87:1004-11. 

10. Under Table 1, I wish there were estimates for intermediate years. I think the authors have not optimally utilized the data. A trend analysis would be useful as well. 

**Response:** The trend between 2010 and 2017 is unlikely to have been linear and would also be hampered by large confidence intervals due to few data points by year. Notwithstanding that, we aimed to provide only the overall difference between 2010 and 2017 in the current work. The predictions are available between the principle prediction years and the data can be obtained for those interested from the primary author (MGC).

11. Can the authors explain the small changes in some districts; and also, why there is such a wide variation in the changes by district? 

**Response:** The changes observed by district are a function of covariates such as interventions, urbanization, land use etc. We did not assemble all possible covariates by district to allow direct comparison by district in a plausibility framework due to data limitations. The wide variation is likely due to sparse data and prevalence within a district spanning different endemicity levels (heterogeneity within districts) and this will be reflected in the Confidence Bounds of each prediction.

**Discussion:**

12. Can the authors discuss the design variation for the different multiple sources of data; 

**Response:**  For national household surveys, these are provided in the links to national household survey designs. For other published works, these are often provided in detail in the peer reviewed publications. This often lead to contacts with the authors for finer spatial and temporal level data not provided in the publication. Other data has been provided without the context of a publication by malariologists working in Malawi, and all acknowledged in the paper. Overall, our only requirement was that a community or a school was sampled as a single cross-section and that information was available on the date, number examined and numbers positive. It is beyond the scope of these large data assembly approaches, levering all possible national survey data, to build a detailed sampling strategy per data point. This is possible when ONLY using multi-stage, household sample surveys. And some investigators only use these for mapping work, building in survey weights. However, it is our view that for NMCPs, these are often limited in the information they can provide on malaria prevalence and ignore the vast amounts of other unpublished works that exist within a country. Here we have levered all possible data from all possible sources to be able to provide the most complete source of data on malaria infection prevalence in Malawi.

13. One of the references has same title as the manuscript.

**Response:** The reference similar to the title of the manuscript references the dataset used in the analysis for future citations where the data provided will be used elsewhere.

14. Do we have any reference to climatic changes within the period of analysis?

**Response:** We have added a reference on the climatic changes within the study period.

Once again, we thank the reviewer and offer the improved manuscript based on this and two other reviews.

# Peer review: Reviewer response for version 1

**DOI:** 10.21956/wellcomeopenres.16576.r35161

**Published:** 2019-05-02

**Authors:** Rocco Panciera

**Affiliations:** 1 United Nation Children’s Fund (UNICEF), New York, NY, USA

The article presents a geostatistical analysis of the temporal and spatial variation of malaria incidence estimated by assembling a national and multi-temporal dataset of malaria prevalence surveys in Malawi between 2010 and 2017. Geostatistical spatio-temporal models are applied to provide granular estimates of malaria prevalence standardized to age 2-10, which are then weighted by population density to estimate changes in malaria prevalence within the period and for all districts in Malawi. Such changes are attributed to Malaria-specific interventions undertaken in the same period under the National Malaria Strategy 2011–2015.

 The paper is well written, scientifically sound and presents conclusions of relevance to inform the success of intervention and their variability across the country. Some comments are provided below in relation to some weaknesses in specific areas.

Are sufficient details of methods and analysis provided to allow replication by others?

- Authors should clarify whether the 10% cross-validation exercise was repeated for multiple extractions.

If applicable, is the statistical analysis and its interpretation appropriate?

- Could the author clarify whether the model produces an estimate uncertainty surface as usual for geostatistical models, and whether this was seen to vary significantly across space (and time), and how would this might affect the conclusion drawn on sub-national variation in rates of decrease in prevalence for particular districts.
- Is it possible that model performance as measured based on 10% hold-out dataset through might vary throughout the temporal, window of analysis 2010-2017?
- When discussing district-level temporal changes in prevalence, authors might want to indicate when the CI includes the null (i.e., in the case of Phalombe district, a decrease of incidence is also compatible with the data (i.e., lower CI of -12%)).
- Authors should comment on how the MAE of 15% might impact the district level changes observed in Table 1.

Are the conclusions drawn adequately supported by the results?

- The Authors claim that no significant climate anomalies where observed during the analysis window (2010-2017). However the work they cite to sustain this claim seems to refer to the previous decade (2000 to 2010). Could a more recent reference relevant to the period at hand be found?
- Other factors, such a socio-economic factors, education, access to improved water and sanitation, and proximate determinants including access to health services are known to affect malaria-related morbidity. The authors should comment on whether such factors might be associated to prevalence rate, and to the extent possible provide argument against their potential impact on the observed reduction

I confirm that I have read this submission and believe that I have an appropriate level of expertise to confirm that it is of an acceptable scientific standard, however I have significant reservations, as outlined above.

# Response

**Published:** 2019-06-19

**Authors:** Michael Give Chipeta

**Affiliations:** Malawi Liverpool Wellcome Trust, Malawi

We thank Dr Rocco Panciera for thoughtful and helpful comments made on the first version.  We have improved the clarity of the paper by responding to the points raised as follows: 

1. Authors should clarify whether the 10% cross-validation exercise was repeated for multiple extractions.
**Response**: The 10% cross-validation exercise was repeated for multiple extraction simulations to ensure that Monte-Carlo error was reduced as much as possible.
2. Could the author clarify whether the model produces an estimate uncertainty surface as usual for geostatistical models, and whether this was seen to vary significantly across space (and time), and how would this might affect the conclusion drawn on sub-national variation in rates of decrease in prevalence for particular districts.
**Response**: The model produces standard error maps, and the 2.5 and 97.5 quintiles. The standard error ranged from 0.0005 to 0.3 across space and time and was largely influenced with availability of data across space and time. Areas with more data were seen to have low standard errors and narrow CIs, Thus, for any policy relevant work, the, managers would be confident in areas with more data and add more data collection initiatives where data were sparse. Additionally, we have added a map of exceedance and non-exceedance probabilities to quantify with 80% or 90% certainty that a location is above or below a given prevalence threshold.
3. Is it possible that model performance as measured based on 10% hold-out dataset through might vary throughout the temporal, window of analysis 2010-2017?
**Response**: While temporal variation would be expected, the amount of data by year is not enough for the exercise. 
4. When discussing district-level temporal changes in prevalence, authors might want to indicate when the CI includes the null (i.e., in the case of Phalombe district, a decrease of incidence is also compatible with the data (i.e., lower CI of -12%)).
**Response**: We have now indicated explicitly in the discussion that changes in the CIs in Machinga, Mulanje and Phalombe districts included the null value.
5. Authors should comment on how the MAE of 15% might impact the district level changes observed in Table 1.
**Response**: The mean absolute error (MAE) represents the average magnitude of the errors (the absolute differences between the predictions and actual the observations). As this is an average measure, some data points with larger values in MAE may have contributed to the value obtained. However, if the interpretation is made at district level, changes ranging between 0 and 15 % would be assumed to be within the MAE bounds and thus null. The correlation was 0.72 indicating a good correspondence between observed and predicted values.
6. The Authors claim that no significant climate anomalies where observed during the analysis window (2010-2017). However, the work they cite to sustain this claim seems to refer to the previous decade (2000 to 2010). Could a more recent reference relevant to the period at hand be found?
**Response**: We have now updated the reference to a more recent one.
7. Other factors, such a socio-economic factors, education, access to improved water and sanitation, and proximate determinants including access to health services are known to affect malaria-related morbidity. The authors should comment on whether such factors might be associated to prevalence rate, and to the extent possible provide argument against their potential impact on the observed reduction.
**Response**: Our modelling framework did not use covariates (such as socio-economic, climate, land use, ecology and interventions) during the spatial-temporal modelling. This is so because we regard parasite prevalence observed at each location and time reflects the effects of all climate, land-use, urbanization and interventions at the time a survey is undertaken. In addition, if these covariates were to be used, we do not share the view that the right data exist, or the right directionality is presumed of covariate selection over-time. The inclusion of intervention covariates introduces a circularity that often leads to an underestimate of predicted P *f*PR, for example P *f*PR in 2017 in a given location is a product of the use of LLIN in that location, to include a covariate that adjusts this value according to LLIN coverage data from other sources seems inappropriate. For these combined reasons we have increasingly defaulted to using the empirical data on parasite rate without covariates, enabling us to more reliably understand the likely impacts of time-varying environmental and intervention effects at a continental (Snow *et al*., 2017), national (Macharia *et al.,* 2018; Giorgi *et al.,* 2018) and sub-national scale (Snow *et al*., 2015) using a qualitative plausibility framework. 

Giorgi E, Osman AA, Hassan AH, Ali AA, Ibrahim F, Amran JGH, et al (2018). Using non-exceedance probabilities of—relevant malaria prevalence thresholds to identify areas of low transmission in Somalia. Malar J., 17:88

Macharia PM, Giorgi E, Noor AM, Waqo E, Kiptui R, Okiro EA, Snow RW (2018). Spatio-temporal analysis of *Plasmodium falciparum* prevalence to understand the past and chart the future of malaria control in Kenya. Malaria J., 17: 340

Snow RW, Sartorius B, Kyalo D, Maina J, Amratia P, Mundia CW, Bejon B, Noor AM (2017). The prevalence of *Plasmodium falciparum* in sub Saharan Africa since 1900. Nature, 550: 515-518

Once again, we thank the reviewer and offer the improved manuscript based on this and two other reviews.
